# Rhode Island Domestic Violence Shelter Policies, Practices, and Experiences Pertaining to Survivors With Opioid Use Disorder: Results of a Qualitative Study

**DOI:** 10.1177/1178221818812895

**Published:** 2018-11-27

**Authors:** Emily F Rothman, Rebecca Stone, Sarah M Bagley

**Affiliations:** 1 Department of Community Health Sciences, School of Public Health, Boston University, Boston, MA, USA; 2 Sociology Department, College of Arts and Sciences, Suffolk University, Boston, MA, USA; 3 Departments of Medicine and Pediatrics, School of Medicine, Boston University, Boston, MA, USA; 4 Grayken Center for Addiction, Boston Medical Center, Boston, MA, USA

**Keywords:** Opioid use disorder, intimate partner violence, domestic violence, shelter, qualitative

## Abstract

The purpose of this study was to fill a gap in the existing research by exploring policies and practices of domestic violence shelters in one US state pertaining to clients with opioid use disorders (OUDs), as well as documenting some of their experiences providing services to those clients. We used semi-structured key informant interviews to gather information about Rhode Island shelter practices and policies pertaining to OUD-relevant topics and make meaning of shelter workers’ experiences with clients with OUDs. One researcher conducted all 30-min, telephone interviews. Qualitative data were analyzed using a content-based analysis approach. The open-ended interview questions yielded information that clustered in the following three main themes: (1) existing shelter policies and practices; (2) staff training on OUDs; and (3) ideas for improvement. Intimate partner violence (IPV) service providers reported that OUD is an issue that affects their clientele, creates problems for both IPV survivors and for staff who are helping them heal from IPV, and causes concerns about the safety of children and other shelter residents who may be housed with individuals with OUDs. Participants reported a range of policies and practices related to how IPV survivors with OUDs are served by their programs. They also offered multiple possible improvements that could be made to IPV survivor programming. Among their suggestions were the establishment of long-term housing, hiring substance use disorder specialists to work in IPV shelters, and improving interagency relationships between IPV programs, child protection services, and substance use disorder treatment providers. Some close-ended interview questions permitted calculations about the percentage of programs that had particular policies in place. For example, of the six programs, 50% (n = 3) reported that they keep naloxone on site. Only one of the six programs (18%) reported that they have a protocol for disposing of unused opioids, medications for OUD, or drug paraphernalia if it is found at the shelter. Additional data about the prevalence of OUDs among the IPV shelter population is needed, as are in-depth analyses of barriers and facilitators to OUD treatment for IPV survivors.

## Background

Opioid use disorder (OUD) is a major public health concern in the United States, and confronting the opioid epidemic has become a top public health priority. In 2016, there were 42 249 opioid-related overdose deaths in the United States, or 13.3 per 100 000 persons.^1^ This death rate represents a 28% increase from 2015.^1^ On average, 115 Americans die every day from an opioid overdose.^2^ Emergency department visits for opioid overdoses rose 30% in the United States from July 2016-September 2017.^3^ Importantly, in the United States, women are increasing heroin use at a faster rate than men, but decreasing nonmedical prescription opioid use at a slower rate than men,^4^ so there is a particularly urgent need to understand barriers to OUD treatment for women.

Intimate partner violence (IPV) is also a major public health concern. According to the U.S. Centers for Disease Control and Prevention (CDC), approximately 36% or 43.6 million US women have experienced sexual or physical violence, or stalking, by an intimate partner during their lifetimes.^5^ More than 5% (5.4%) of US women have these events occur in a 12-month period. Intimate partner violence has numerous health consequences. Affected women are three to five times more likely than other women to exhibit post-traumatic stress disorder (PTSD), depression, suicidal ideation, and substance use.^6^ Around 42% of women who have experienced IPV sustain injuries.^7^

There is substantial overlap in IPV victimization and OUD morbidity; women who experience IPV are at increased risk of OUD. A recent nationally representative study found that IPV survivors were at 24 times the risk for OUD as those with no IPV history (2.4% vs 0.1%)^5^ and that female survivors of IPV were at three times the risk for OUD as compared with male IPV survivors.^8^ There are several reasons why IPV survivors may be at increased risk for OUD. Between 51% and 75% of IPV survivors experience PTSD, and people with PTSD have 60% increased odds of developing an OUD than those without PTSD.^9^ Substance use is a common coping mechanism for IPV survivors.^10,11^ Some IPV survivors are encouraged or forced to take opioids by abusive partners who want to keep them docile, dependent, or want them to appear irresponsible to the court in custody cases.^11^ Not only is IPV associated with the risk for subsequent OUD, but drug use has also been associated with increased risk of IPV victimization.^5,6^ This suggests that the relationship between OUD and IPV may be bidirectional and reciprocal. Furthermore, partners’ tactics of controlling access to drugs,^12^ as well as the stigma associated with drug use, can increase relationship dependencies and complicate efforts to leave the relationship.^13,14,15^

There have been calls for better collaboration between domestic violence and substance use treatment providers in general for more than a decade.^14^ However, a previous survey of IPV shelters in one state found that only half of the programs had policies related to clients who use substances, and only one-fourth had memoranda of agreement with substance use treatment providers.^16^ Research in both rural and urban communities has indicated that women face significant affordability, availability, and accessibility barriers to health and mental health service use and that women with victimization histories face additional barriers more than women without such histories.^7^ Working with IPV survivors with OUDs requires specific knowledge about responding to overdoses and facilitating access to OUD treatment, so focusing on improving collaborations between OUD treatment providers and IPV programs, specifically, makes sense. Despite recognition that IPV programs could, and should, be addressing the substance use related needs of survivors more comprehensively, there has been little research on this topic to help guide shelter workers in developing better policies. Dr Carole Washaw, the Director of the National Center on Domestic Violence, Trauma & Mental Health, has provided webinars and other trainings to IPV services providers on the intersection between IPV and OUDs that, anecdotally, have been very helpful to the community of IPV service providers, but too few published, peer-reviewed articles are available on the topic.^8^

The purpose of this study was to fill a gap in the existing research by exploring policies and practices of domestic violence shelters in one US state pertaining to clients with OUDs, as well as documenting some of their experiences providing services to those clients. Note that although the term “intimate partner violence” is more commonly used by researchers in research reports, the term “domestic violence” is still in use in the field (eg Rhode Island Coalition Against Domestic Violence [RICADV]). Therefore, both terms are used interchangeably in this article.

## Methods

The study design was exploratory and descriptive. We used semi-structured key informant interviews to gather information about Rhode Island shelter practices and policies pertaining to OUD-relevant topics, and make meaning of shelter workers’ experiences with clients with OUDs.

### Recruitment and sample

The first author attended an in-person meeting of the executive directors (EDs) of RICADV programs in February 2017. The five EDs of the five programs that are full members of the RICADV were present, as well as one ED of one of the four partial member programs. All EDs present at the meeting were asked if they would consider participating in a study about IPV survivor clients and OUDs. All six EDs expressed support for the research project during the meeting with no deliberation. Subsequently, the research team submitted a protocol to the institutional review board (IRB) of the first and third authors. The research was determined to be exempt. Next, the first author emailed the EDs who had been present at the February meeting to ask them if they would schedule interviews. All six EDs responded that telephone interviews could be arranged with them or a representative of their program. Therefore, the inclusion criteria were either (1) being the ED of one of the five full member programs of the RICADV, or one of the partial member programs, or (2) being the ED’s designee. In two out of six cases, EDs nominated designees because they felt that a different program staff person had more knowledge about the topic of OUDs among program clients that they did themselves. The sample size was considered appropriate because it was comprehensive of RICADV full member programs, the study was not funded (ie resources for recruitment and interviews were limited), and because this was an exploratory, qualitative study.

### Data collection

The first author conducted all six interviews over the telephone. Each interview lasted approximately 30 min. Five interviews were one-on-one, and one interview included one ED and two additional staff members of an IPV program. The interviewer took detailed notes during the interviews on a computer and typed quick enough to capture word-for-word quotations for most responses. In instances when the key informant made a comment that the interviewer anticipated might be particularly germane, given the research questions of interest (eg telling a story about an opioid-related overdose or describing a policy), the key informant was asked to repeat the comment slowly so that the interviewer could capture it precisely. The comment was then read back to the key informant to verify that their statement had been captured accurately.

The interviewer used the same interview protocol (ie set of interview questions) for each interview and asked the questions in the same order. Most questions were open ended, although there were five closed-ended questions asked to obtain some factual, programmatic information (presented in Table 1). An example closed-ended question was: “Do you keep any Narcan or naloxone on the premises?” Because none of the programs keep data about how many clients have an OUD or struggle with opioid use, each key informant was asked to estimate the percentage of program clients who were struggling with an OUD, and the estimated percentage who were receiving methadone treatment while being an IPV shelter resident. In keeping with best practices in qualitative research, the interviewer probed when illuminating comments were made and asked impromptu questions when relevant sub-topics were spontaneously introduced by the key informant.^9^ Sample interview protocol questions included: “What anecdotes or stories can you share about clients and opioid use as ‘lessons learned’ for the field?” “What are your personal feelings about substance using-survivors remaining in shelter and/or continuing to receive services?” and “If you could have all the money in the world, and do anything you wanted to improve conditions for your program clients in terms of opioid use problems, what would you do?”

**Table 1.** Opioid-related characteristics of Rhode Island domestic violence shelters (N = 6).

Characteristic	% (N)
IPV program characteristics	
Number of employees (range)	8-31
Number of shelter bed nights per year (range)	1395-6970
Opioid use–related information	
Estimated % of past-year clients with opioid use disorder (range)	12-30%
Estimated % of past-year clients on methadone during shelter stay (range)	0-40%
Program asks clients about substance use during intake interview	66% (4)
Program does not shelter IPV survivors with substance use disorders who are not in recovery (ie “dry shelter”)	33% (2)
Program has ever had a client overdose while a shelter client	33% (2)
Program keeps naloxone on site	50% (3)
Program has a protocol for disposing of unused opioid medication, methadone or drug paraphernalia	18% (1)
Program keeps data records on the number of clients with substance misuse	0% (0)

IPV: intimate partner violence.

### Analysis

Responses to closed-ended questions were tallied and are presented in table format (see Table 1) to characterize the programs. Qualitative data were analyzed using a content-based analysis approach. First, the texts of all interviews were read by the first and second authors to “get a sense of the whole.”^10^ Next, these two research team members generated codes, agreed upon the codes, and sorted the text into categories by code. The data analysts reviewed the sorted text, identified themes, and selected illustrative quotations that exemplified each theme for presentation.

## Results

Two of the six programs reported that they have a policy to ask prospective clients about their substance use during an intake interview and do not house those who are using opioids and not engaged in some type of treatment. Program staff were asked to estimate to the best of their knowledge the percent of past-year clients who had struggled with OUD; estimates ranged from 12% to 30%. The staff were also asked to estimate the percentage of past-year clients who were receiving methadone or another opioid treatment medication such as buprenorphine or naltrexone, during their shelter stay; estimates ranged from 0% to 40%. Two staff reported experiences when an IPV program client overdosed on opioids while a resident of the shelter, and two reported hearing about an IPV program client overdosing after leaving the shelter program. No opioid-related deaths were reported in the shelters themselves, but two staff did recall former clients who fatally overdosed after leaving the shelter. Of the six programs, 50% (n = 3) reported that they keep naloxone on site. Only one of the six programs (18%) reported that they have a protocol for disposing of unused opioids, medications for OUD or drug paraphernalia if it is found at the shelter. None of the programs reported that they track the number of their clients who have substance use disorders.

The open-ended interview questions yielded information that clustered into that following three main themes: (1) existing shelter policies and practices; (2) staff training on OUDs; and (3) ideas for improvement. Each theme is presented in greater detail below.

### Theme 1: shelter policies and practices

The main sub-topics related to shelter policies and practices pertained to (1) whether or not programs assessed prospective clients’ substance use disorder needs at program intake, (2) why policies about barring people with OUDs from shelters had evolved over time; (3) whether programs made referrals to inpatient or outpatient programs for clients with OUDs; and (4) how programs confronted challenges related to rural isolation and transportation on behalf of clients who needed to get to OUD treatment.

Key informants prefaced their comments about program policies and practices with some discussion of whether OUD were prevalent among their service population, and most noted that they had observed an increase in OUD among clients recently. A common theme was that there had been a recent increase in the number of clients who needed or deserved more support for OUD and that they were anxious and upset by the extent to which client need for quality substance use disorder services outweighed what was available. In the words of one program director,

I’m 30 years into this movement, and in the past 10 years this has become a big problem. It had stopped for a minute and now we’re seeing it again. It is so difficult for [the clients] to stay sober. I’ve had many sleepless nights trying to get them through.

A particular concern about under-attention to the issue of OUD in IPV survivor clients was that Black women receive disproportionately less support. As one program staff person explained, she was concerned that staff could be quicker to identify substance use disorder problems in White and Latina clients, but overlook the needs of Black women clients because “there is a stereotype that Black women are strong so [they] don’t get offered the services that they need.”

One recurring theme was that programs did not take a uniform approach to the question of whether or not to ask about substance use during a shelter intake interview, and the programs had clearly weighed pros and cons of their practices. Two shelters reported no screening policy at intake. This was the case even for one shelter that explained that they are “technically a dry shelter, but we don’t have substance abuse as a screening question when they check in.” The other shelter commented: “We do not have a policy to screen new clients [for substance use], because policies can create an unhealthy behavior where there is no wiggle room.” This program, like most others, reported that they handle cases “situation by situation.” In part, decisions about whether or not to shelter, or to continue to shelter, clients who are actively using substances depend on whether there are minor children also residing in the shelter and if the client’s substance use could conceivably cause harm to others (ie leaving needles in a room).

Other shelter programs reported a more flexible approach that had evolved over time. For example, one stated that at one time, they had a policy that clients “had to be clean,” but that they now allow clients with OUD and other substance use disorders to remain in their shelter if they were enrolled in an outpatient treatment program and not actively or obviously using (*NB*: “clean” is a slang term for substance-free that is not used by treatment providers, but is used here to preserve the integrity of the data). Other programs had gone through similar evolutions in their approach, and as one staff person explained,

It used to be that we took a real hard line on it, that you had to be clean for six months, and I think that’s a really unrealistic approach because the people we serve are in crisis. So we’ve relaxed our rules … That approach was unrealistic because it encourages clients to lie to us and we want to get them the help that they need … about 10-15 years ago was the beginning of starting to relax rules a little bit. We went from [a requirement of having been] six months clean to three months clean and then down from there. It’s mostly about safety for other clients living there.

Another informant explained that their agency’s rationale for using a harm-reduction approach was to provide the maximum amount of support to IPV survivors. She said,

You have to meet clients where they are, and if they are actively using substances they can still benefit from support we can provide them. It’s more on us to figure out how we can make our services accessible. I am always thinking how can we reach more clients, not how can we exclude clients.

Programs generally did not have any specific rules or policies about making referrals to OUD treatment programs. Whether referrals were made seemed in some cases to depend on whether the IPV program staff had established relationships with staff of local substance use treatment provider agencies. These agency-to-agency relationships were reportedly more difficult to maintain in recent years, as substance use disorder treatment providers have struggled with burgeoning caseloads and decreases in funding. In the words of one staff person,

The days of having a set relationship with an inpatient program are over. I do have an excellent relationship with [an outpatient addiction program] and I check in with them every three months to make sure they are still there. Back in the day we had those relationships, but the [substance use treatment] programs shut down.

Other directors explicitly mentioned the lack of community OUD treatment services for women as a major obstacle to their health and well-being. One program staff person said that she was sure “we have had clients who were willing to get treatment but couldn’t get it because of no free slots.” A different staff person explained why it is particularly difficult for IPV shelter clients to get the OUD treatment that they need:

There are not enough substance abuse places. It takes 1-2 weeks to even get an assessment, and then inpatient would take longer and if you don’t have insurance you are out of luck. If would take an extra month to see someone on an outpatient basis for treatment. We really have not made any formal connection or relationship with any agency in addiction treatment or addiction medicine. If we did, and had a contract, it would be nice if we could get the women in immediately.

Staff located in rural areas, where treatment providers are few and far between, expressed even more acute frustration about the lack of OUD treatment services: “We are in [one of the most] … rural parts of the state. We have one mental health counselor for all of [this county].”

More than one staff person mentioned that transportation was one of the most significant barriers that IPV survivors who wanted OUD treatment such as methadone had faced. Some program staff reported that their shelters support clients by providing transportation or money for the bus, but others lamented that they had no transportation support to offer. One staff person explained that they talked to prospective clients during the intake interview about the geographic location and potential problems it would raise for those needing methadone:

We ask patients to be honest with us because we say they are located where they may not be able to get to their maintenance program. The bus may not get there on time on weekends […] If you are a mom with children, and you need to get there Sunday morning, you have to leave here and go somewhere else and take the bus back because there is no crosstown bus and you have to cross four lanes of traffic with kids.

### Theme 2: staff training

On the whole, program staff identified a need for more staff training on OUD and other substance use–related topics but also discussed barriers that deterred them a bit. One problem is that shelters typically employ staff who work various shifts throughout a 24-h period, so it is virtually impossible to bring all staff together for a site training. In the words of one staff person,

We have 24-hour staff so it’s really difficult to get everyone to training, hard to get evening or weekend staff to come in during the week for training.

Another staff person started to explain that staff training was not needed because OUD were not prevalent among clients at her program, but changed her thought mid-stream and ended by acknowledging that there were clients who used opioids in the recent past and that staff training was probably needed. She said,

We really haven’t had training on this because we haven’t seen it a lot. If it were something staff came to me about and said we need training on this then we would address it, but … we are trying to be a little but more proactive and we have had a couple of clients [who were using opioids].

Three key informants reported that their staff had received specific training to recognize the signs of OUD overdose and how to administer Narcan (ie naloxone). One additional staff person said that her agency would be participating in a Narcan training in the near future and that they did not presently keep Narcan on site. Of note, two of the six programs had been able to have staff participate in a Narcan training through a state Department of Health program, and the fact that the trainings were free to the IPV programs was a major factor in their decision to participate. As one program staff person said: “Anything free regarding stuff going on in the world, I’m there.”

Two programs do not keep Narcan on site and have not yet trained staff to administer it. They had reasons why this was the case. One program said that staff were encouraged to call 911 for all emergencies, including opioid-related overdoses, for safety reasons. She was worried that they might administer Narcan incorrectly even after training and that there could be liability issues or other problems because, in her words: “We aren’t medical professionals.” This sentiment was echoed by another research participant, who explained “We are not supposed to dispense aspirin, so talk about assisting someone who is in overdose! I wonder about the legality of that? We’re not nurses.”

### Theme 3: improving IPV shelter services for clients with OUDs

Key informants were asked to reflect on their experiences providing direct service to IPV survivors who had OUDs. Each expressed deep empathy for program clients and were quick to point out the myriad ways in which coping with an OUD could present additional challenges and/or dangers. One key informant located in a non-urban area reported that the relative lack of OUD treatment providers was a particular concern because of the potential for the survivor to see the perpetrator of IPV at the treatment provider agency. In her words,

It can be dangerous for women who have to leave the shelter to get methadone or go to a clinic because those types of places are limited nearby, and so if she was using with her abusive partner, she could run into him there. One woman told me, “he saw me, he chased me.” I had to take one survivor, she was so scared, to get her methadone. So even if they are trying to get well, there is this other piece of danger.

Thus, a primary recommendation for improving services for IPV survivors with OUDs was to facilitate transportation to OUD treatment agencies, and to do so in a way that took into account the potential danger that IPV survivors face when they need to go in-person to a treatment location.

A second recurring theme was that program staff worried about children’s safety in shelters where opioid-using clients were permitted to stay, and wished that there were separate transitional housing programs for IPV survivors in recovery. In the words of one key informant,

It is challenging when you have children in the shelter and [you are] running a harm reduction [model]. Sometimes the actively abusing person is high or drunk at the shelter and acts in a way that is not adult-like, not causing harm but swearing and yelling and engaging in behavior that is disruptive, and that does seem more complicated when there are small children.

Another solution informants proposed was building more interagency relationships, and as a community ensuring a greater variety of services to help “meet clients where they are.” Several stressed that permanent housing rather than short-term shelters would be beneficial. In the words of one informant,

I would have more housing. I am not a big fan of “shelter” because everyone needs permanent housing in the long run, so affordable housing should be the goal.

Another expressed an almost identical sentiment:

I would eliminate the concept of shelter if I could. I would look at someone on the road to recovery … and give her permanent supportive housing with support services including relapse prevention, [IPV] education and knowledge, all those components. When you are an addict, it’s severe and shelter is temporary.

Multiple informants also said that having trained substance use disorder counselors on staff at the IPV program would be ideal. Finally, all of the informants reported that they saw stronger interagency relationships as essential to improved responses to IPV survivors with OUDs in the future. There have been many gains in recent decades in terms of how IPV programs, substance use treatment programs, and state agencies work together, but informants felt that there was still a need to work on creating even stronger partnerships. In the words of one informant,

The piece that is really concerning is that there is still a breakdown in communication with [child protective services] and the [intimate partner violence] community and the substance abuse community.

## Discussion

This research resulted in several novel findings for both the IPV and OUD prevention fields. First, IPV shelter providers reported that OUD is an issue that affects their clientele, creates challenges for both IPV survivors and for staff who are helping them heal from IPV, and causes concerns about the safety of children and other shelter residents who may be housed with individuals with OUD. Second, participants reported a range of policies and practices related to how IPV survivors with OUD are served by their programs. Uniformity across programs may not be a necessary or worthwhile goal, but understanding the rationales for particular approaches and potentially best practices with regard to several aspects of IPV survivor services would benefit the field. Specifically, information about the effect of screening or not screening prospective clients for OUD at program intake would be useful, as well as information about whether and how referrals to OUD treatment are made, and assessments of the impact of training IPV program staff about OUD and related topics such as the use of naloxone or safe disposal of opioids and drug paraphernalia are urgently needed. Third, participants in this research offered multiple insightful possible improvements that could be made to IPV survivor programming. Among their suggestions were the establishment of long-term housing; hiring substance use disorder specialists to work in IPV shelters; and improving interagency relationships between IPV programs, child protection services, and substance use disorder treatment providers.

Additional observations about the findings are that all the IPV program providers expressed respect for individuals with OUD, and each one articulated reasons why recovering from IPV can be more complicated for those with OUD (and vice versa). In cases where some reluctance about additional staff training on OUD was detected, there was a sense that it was because IPV program staff are already overburdened, overwhelmed and have lengthy lists of types of additional training that would be beneficial (eg changes to immigration law and asylum-seeking by IPV survivors, HIV prevention medication options, and the connection between IPV and human trafficking). When EDs need to be make choices about how to prioritize topics for training staff or forging new community-based interagency alliances, they are forced to rank-order the most pressing impediments to the health, safety, and welfare of their clients—whether OUD will be at the top of the list in the years ahead is unclear, in part because we lack data about how prevalent OUD is among the IPV shelter client population.

Shelter workers’ estimates about the prevalence of OUD among their clients are unlikely to be sufficiently accurate for intervention planning or staff training purposes. To our knowledge, there are no existing estimates of opioid use among the IPV shelter population for any state, let alone the nation. Studies of the prevalence of IPV among women who are using methadone or are in substance use disorder treatment do not answer the questions that the IPV service providers need answered: is OUD a large or small problem in the IPV program client population? Which IPV program clients are most at risk for OUDs? And which policies, practices, and service models tend to result in the best outcomes for those individuals and, if relevant, their children? These are critically important research questions that remain to be answered. Given the nature and scope of the opioid epidemic in the United States, ideally resources will be devoted to research that provides answers to these questions without delay.

This study was subject to several limitations. Qualitative research is not intended to be broadly generalizable beyond a specific context.^11,12^ Nevertheless, this study included representatives of every full member program of the RICADV to gather the spectrum of opinions and concerns from every type of Rhode Island IPV shelter program. These results may not be representative of IPV programs in other US states, nor in other countries. Additional research that attempts to expand upon these findings in other states is needed. Second, qualitative research is inherently subjective. In this case, the questions that were asked of the IPV programs were relatively straightforward so the risk that the perspectives and opinions of the research team influenced findings adversely is relatively low. Third, not every staff member of every IPV program was interviewed. It is possible that the memories, opinions, experiences, and ideas of these four EDs and their two designees do not reflect the thoughts of other IPV program staff people. This possibility could be investigated through a larger-scale study that incorporates data from more than one person per program. An additional limitation of this study is that our focus is on violence against women and shelters that serve predominantly women and children. As described in the introduction, women who experience both IPV and OUD are underserved and face many barriers to accessing the few resources that do exist. It is also true that male survivors of IPV are drastically underserved in most communities and that there are even fewer shelters and other service providers for this population. There is a great need for further research into the needs of male IPV survivors with substance use disorders.

In conclusion, this exploratory and descriptive study is intended to break new ground in the intersection between IPV program service provision and OUD treatment services research. To our knowledge, it is among the first to explore themes related to IPV program policies and practices pertaining to clients with OUDs, and it lays a foundation upon which additional, larger-scale, and more complex research studies should be built.
